# A Multi-Agent and Attention-Aware Enhanced CNN-BiLSTM Model for Human Activity Recognition for Enhanced Disability Assistance

**DOI:** 10.3390/diagnostics15050537

**Published:** 2025-02-22

**Authors:** Mst Alema Khatun, Mohammad Abu Yousuf, Taskin Noor Turna, AKM Azad, Salem A. Alyami, Mohammad Ali Moni

**Affiliations:** 1 Institute of Information Technology, Jahangirnagar University, Savar, Dhaka 1342, Bangladesh; alema.pgr2017@juniv.edu (M.A.K.); yousuf@juniv.edu (M.A.Y.); 2 Department of Information and Communication Engineering, Pabna University of Science and Technology, Pabna 6600, Bangladesh; taskin.it1405@pust.ac.bd; 3 Department of Mathematics and Statistics, Faculty of Science, Imam Mohammad Ibn Saud Islamic University (IMSIU), Riyadh 13318, Saudi Arabia; kazad@imamu.edu.sa; 4 King Salman Center for Disability Research, Riyadh 11614, Saudi Arabia; saalyami@imamu.edu.sa; 5 Artificial Intelligence & Data Science, School of Health and Rehabilitation Sciences, Faculty of Health and Behavioural Sciences, The University of Queensland, St Lucia, QLD 4072, Australia; 6 Rural Health Research Institute, Charles Sturt University, Orange, NSW 2800, Australia; 7 Artificial Intelligence and Cyber Futures Institute, Charles Sturt University, Bathurst, NSW 2795, Australia

**Keywords:** disability assistance, artificial intelligence, deep learning, machine learning, activity recognition, CNN, BiLSTM, attention

## Abstract

**Background:** Artificial intelligence (AI)-based automated human activity recognition (HAR) is essential in enhancing assistive technologies for disabled individuals, focusing on fall detection, tracking rehabilitation progress, and analyzing personalized movement patterns. It also significantly manages and grows multiple industries, such as surveillance, sports, and diagnosis. **Methods:** This paper proposes a novel strategy using a three-stage feature ensemble combining deep learning (DL) and machine learning (ML) for accurate and automatic classification of activity recognition. We develop a unique activity detection approach in this study by enhancing the state-of-the-art convolutional neural network (CNN) and bi-directional long short-term memory (BiLSTM) models with selective ML classifiers and an attention mechanism. Thus, we developed an ensemble activity recognition model, namely “Attention-CNN-BiLSTM with selective ML”. **Results:** Out of the nine ML models and four DL models, the top performers are selected and combined in three stages for feature extraction. The effectiveness of this three-stage ensemble strategy is evaluated utilizing various performance metrics and through three distinct experiments. Utilizing the publicly available datasets (i.e., the UCI-HAR dataset and WISDM), our approach has shown superior predictive accuracy (98.75% and 99.58%, respectively). When compared with other methods, namely CNN, LSTM, CNN-BiLSTM, and Attention-CNN-BiLSTM, our approach surpasses them in terms of effectiveness, accuracy, and practicability. **Conclusions:** We hope that this comprehensive activity recognition system may be augmented with an advanced disability monitoring and diagnosis system to facilitate predictive assistance and personalized rehabilitation strategies.

## 1. Introduction

### 1.1. Background and Motivation

**Figure 1:**
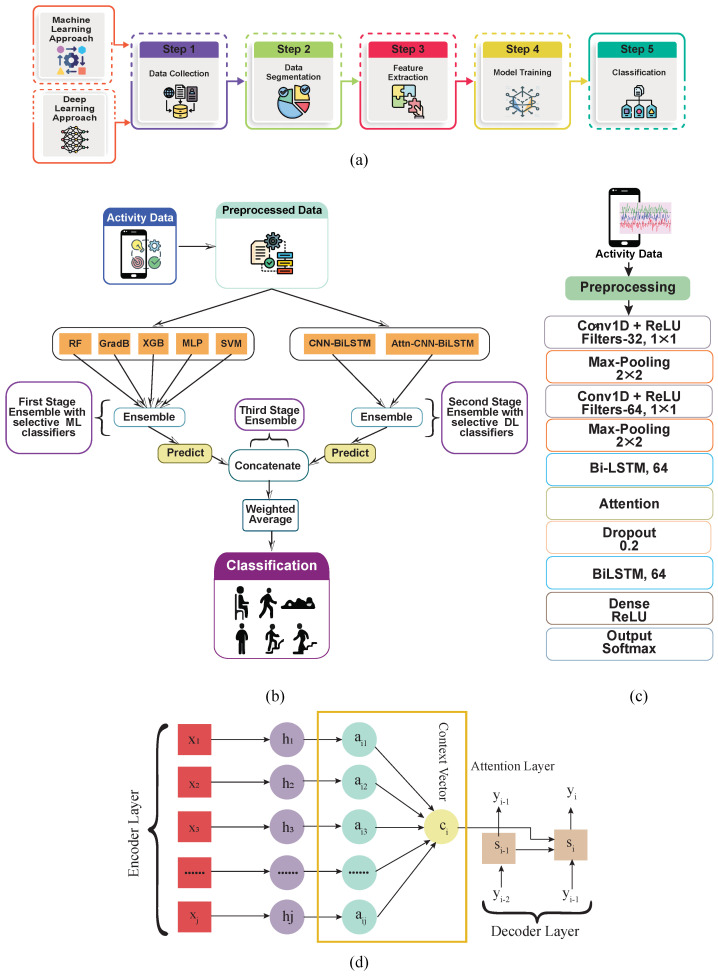
An overview of HAR framework: (**a**) machine learning (ML) and deep learning (DL) approach. (**b**) The schematic diagram of the proposed approach includes preprocessing, classifier selection, ensemble, classification, and verification. (**c**) Proposed Attention-CNN-BiLSTM model. (**d**) The framework of the attention mechanism.

Human activity recognition (HAR) using sensors has become one of the most dynamic and impactful research areas due to its wide-ranging applications in healthcare, sports, smart homes, and human–computer interaction. Using inertial sensors embedded in smartphones and wearable devices, such as gyroscopes, accelerometers, and magnetometers, it is possible to collect time-series data and accurately infer human activities [1]. A typical HAR framework includes five critical stages: data collection, preprocessing and segmentation, feature extraction, model training, and activity classification, as illustrated in Figure 1.

While traditional methods have made significant progress, challenges such as handling diverse activity patterns, mitigating sensor noise, and improving model adaptability remain. For example, in our previous study [2], we introduced a smartphone-based HAR model that employs selective classifiers for better accuracy in specific activities, emphasizing the importance of adaptable systems. Later, in another study [3], we developed a deep CNN-GRU model that leverages automatic feature extraction to achieve real-time recognition, showcasing improvements in both feature representation and adaptability. These studies highlight the crucial role of classifier selection and advanced deep learning architectures in modern HAR systems.

Data preprocessing and segmentation are pivotal to HAR as raw sensor data often contain noise and irregularities. Segmentation into equal-length windows with overlap ensures consistency, while feature extraction—performed using either machine learning (ML) or deep learning (DL)—determines the efficacy of the recognition model. Traditional ML methods rely on handcrafted features in both time and frequency domains, such as mean, entropy, and energy. Although these methods capture specific patterns, they often fail to generalize across similar activities, such as distinguishing between sitting and standing [4]. To build HAR on smartphones, some studies have employed the ML methodology [5,6]. Activity extraction problems in conventional ML can be avoided with the use of DL [7]. Figure 1a illustrates how deep learning (DL) and machine learning (ML) can be applied in human activity recognition (HAR) across different types of systems. The extraction of features and model training procedures are carried out simultaneously using the DL technique, while in the conventional ML technique, the functions must be constructed individually. Instead, they can be acquired proactively throughout the network.

Recent research has extended HAR applications to domains like rehabilitation and pose estimation. For instance, Li [8] developed an in-home lower body rehabilitation system using pose estimation to facilitate precise movement analysis, while Wang [9] optimized datasets and models for rehabilitation, improving motion tracking. Additionally, Wang’s TransNet [10], a parallel encoder architecture, advanced the capture of complex human motions for health-related applications. However, these works lack a comprehensive ensemble model that integrates multiple methodologies to achieve both robustness and high accuracy.

To address these gaps, we introduce a novel three-stage HAR ensemble model, “Attention-CNN-BiLSTM with selective ML”. This model synergistically combines an attention mechanism, CNN, BiLSTM, and selective ML algorithms to enhance prediction accuracy and generalizability. Unlike existing methods, our approach employs weighted aggregation to integrate the strengths of multiple models, achieving robust and adaptable performance across diverse datasets. This innovative framework is particularly tailored to support disabled individuals, offering precise activity recognition that can be applied in healthcare for diagnosis, rehabilitation, and adaptive systems.

### 1.2. Related Work

In the existing literature, various methods for activity recognition have been proposed, categorized as machine-learning-based HAR, deep-learning-based HAR, attention-based HAR, and hybrid-model-based HAR [11]. Over the past decade, researchers have predominantly focused on developing effective human activity recognition (HAR) systems through feature engineering using traditional machine learning approaches. Today, researchers employ deep learning algorithms to classify activities that retrieve sequential and spatial data from an ordered series of frames [12]. Therefore, healthcare, sports, and IoT-based businesses can employ an effective action recognition categorization approach for sophisticated handheld devices. The following sections provide a literature review regarding the relevant existing approaches.

#### 1.2.1. Machine-Learning-Based HAR

Researchers have used a variety of standard machine learning (ML) techniques over the past few decades to identify various human behaviors using data from wearable sensors or cell phones. In one study [13], a HAR system using wearable sensors is proposed, and four publicly available datasets are used. Five machine learning algorithms are tested for their ability to recognise human activity. The gathered results demonstrate that the proposed technique exhibits superior performance in identifying human activities. Garcia-Gonzalez achieved strong smartphone-based HAR results with feature engineering and ML techniques tailored for real-life conditions, but the model lacked scalability across diverse datasets [14]. In contrast, our three-stage ensemble model, integrating selective ML and deep learning, enhances adaptability across varied data sources, effectively managing cross-sensor and cross-environmental variability.

#### 1.2.2. Deep-Learning-Based HAR

Recent advancements in deep learning for human activity recognition have been driven by representation learning techniques, enabling automatic feature extraction from raw sensor data. Paper [15] introduces a method combining 3DCNN with ConvLSTM layers, enhancing traditional 3DCNN performance in HAR. The authors of paper [16] propose four deep learning approaches, CNN-net, CNNLSTM-net, ConvLSTM-net, and StackedLSTM-net or Ensem-HAR, which aggregate predictions for final prediction on test data using a blender or meta-learner. Another study [17] presents CNN-GRU, a hybrid model that outperforms existing models on the WISDM dataset. Wu proposed a new deep learning method that integrates 3DCNN with convolutional long short-term memory (ConvLSTM) layers for HAR and optimizes the traditional 3DCNN [15]. Yalcinkaya [18] and Khan [19] improved HAR for human–robot collaboration and IoT settings by integrating localization and collaboration-specific features, although they lack generalizability, which has been tackled by our proposed three-stage ensemble approach.

#### 1.2.3. Transfer-Learning-Based HAR

Transfer learning, a method in machine learning, involves utilizing a pre-trained model as the basis for a new task. In essence, a model originally developed for one task is repurposed for a similar task, streamlining progress in modeling the second task. A two-component HAR transfer learning framework is proposed in the study [20]. First, a representational analysis identifies features that are user-specific and require customization, as well as features that are common to all users. With this knowledge, they only fine-tune the remaining offline classifier components and transfer the reusable portion to new users. The authors of paper [21] proposed that three steps make up the transfer learning framework: (1) use principal component analysis (PCA) transformation to extract seminal information from the source and target environments; (2) use the Jensen–Shannon divergence (JSD) algorithm to measure feature similarity; and (3) use the Gale–Shapley algorithm to map features to a common space. If the training sample is insufficient for the new environment in which the model is applied, the transfer framework can help improve the recognition performance. It can also lessen the work required to gather labeling data.

#### 1.2.4. Attention-Based HAR

The approach presented in paper [22] combines properties from many convolutional stages to produce a more thorough representation of features. Furthermore, an attention agent is implemented to extract finer details, resulting in improved model accuracy. For identifying human actions in videos, paper [12] introduces a bidirectional long short-term memory (BiLSTM)-based attention mechanism alongside a dilated convolutional neural network (DCNN). This mechanism selectively emphasizes effective features in input frames. By enhancing features that preserve more information through residual blocks, the model extracts salient discriminative features using DCNN layers. These features are then input into a BiLSTM to capture long-term dependencies. In contrast to window-based recognition, a method for classifying all frames was proposed in paper [12]. Multiple convolutional neural networks with varying kernel sizes were used to extract features, which were then fused and used for implementation.

#### 1.2.5. Ensemble-Learning-Based HAR

Activity identification accuracy may potentially be increased with the application of ensemble learning, which aggregates the outputs of several classifiers to get a single estimation result. Minowa [23] proposes an ensemble approach for managing incomplete sensor data, Raj [24] proposes CNN-based HAR improvements, and Zhang [25] shows feature fusion models; all contribute insights into ensemble robustness and accuracy. Our model builds on these efforts, employing a three-stage architecture (ML, CNN-BiLSTM with attention, and final weighted ensemble) for high adaptability and accuracy across varied data sources.

The preceding section covered recent, significant research on human activity recognition that considered deep learning, conventional machine learning, ensemble learning, and hybrid models. According to this literature review, there is a lot of room for experimentation with a multistage ensemble approach. A prevalent constraint identified by examining current models is their deficiency in resilience and generalization capabilities. As far as we know, no prior research has been conducted on the classification of human activity employing a multi- or three-stage ensemble technique. This inspired us to create a brand-new, three-stage ensemble method for classifying human activities using deep CNN-BiLSTM with an attention mechanism and selective ML classifier models. In addition, our study includes a thorough examination of model construction, validation, and selection.

Building upon the limitations and advancements of previous studies in human activity recognition, our research presents significant contributions, which are summarized as follows:

- A novel three-stage ensemble framework that integrates advanced techniques for improved accuracy and robustness;
- An optimized strategy to combine predictions effectively, improving activity differentiation, especially for unbalanced datasets;
- Rigorous evaluation that demonstrates superiority over state-of-the-art methods on WISDM and UCI-HAR datasets;
- A scalable solution designed for healthcare, particularly to assist disabled individuals in rehabilitation and monitoring.

The article is organized as follows. Section 2 represents an overview of the fundamental methods and materials. In Section 3, evaluation and analysis are discussed. Various scenarios for analyzing the accuracy, consistency of our method’s predictions and results are discussed in Section 4. Finally, Section 5 offers reflections on potential future directions.

## 2. Materials and Methodology

Figure 1b shows an overview of the suggested technique with a schematic diagram of the recommended strategy. The first stage is to preprocess the input human activity data before it is supplied to the feature extractor. The procedure of feature extraction is divided into three parts. The first part involves selecting optimum machine learning models and developing an ensemble model. The second stage entails identifying the best individual deep learning (DL) models, leading to another ensemble model’s development. Following that, a third stage of ensemble work is performed utilizing the expected results from the first and second ensembles. For feature extraction, the final ensemble model employs a weighted average layer. The suggested model is validated in terms of many characteristics and experimental settings.

### 2.1. Datasets Description

Activity data is collected through wearable sensors in the form of time series. Raw sensor data serve as the input for activity recognition, with the output being the identified activity class. Our proposed method is validated using two benchmark HAR datasets. Table 1 provides a summary of these public information sources. There are some distinctions between the two. With the greatest number of volunteers, the UCI-HAR dataset was developed using the recordings of thirty participants. The UCI-HAR and the WISDM datasets both have six activities. A summary of the datasets used in our research is provided below.

#### 2.1.1. WISDM

**Figure 2:**
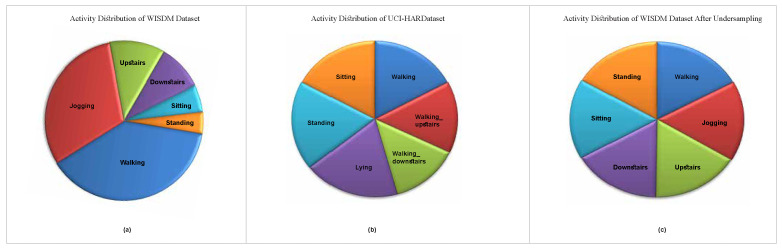
Pie chart representation of the distribution of actions (**a**) of the WISDM dataset including walking, sitting, jogging, descending stairs, ascending stairs, and standing, relative to the 1,086,474 samples in total. According to the data, walking is the most common activity, while standing is the least common. (**b**) Pie chart of the UCI-HAR dataset including walking, standing, sitting, lying down, and going upstairs and downstairs, relative to the 10,299 samples in total. According to the data, lying is the most common activity, while going downstairs is the least common. (**c**) Depiction of the distribution of actions of the WISDM dataset after undersampling to balance the dataset.

The WISDM dataset is a reference for human activity recognition (HAR) and originates from the Wireless Sensor Data Mining Laboratory [5]. It encompasses a total of 1,086,474 individual samples. This dataset focuses on recognizing activities and was amassed from 36 users engaged in their everyday routines. These routines encompass six distinct behaviors: walking, sitting, jogging, descending stairs, ascending stairs, and standing. Participants were involved in data collection by carrying an Android smartphone in the front pocket of their trousers. The phone’s built-in accelerometer sensor was utilized to record the data, capturing movements at a frequency of 20 Hz. From Figure 2a, it is evident that the dataset in WISDM is not balanced. Walking comprises 38.6% of the activities, while standing accounts for only 4.4%.

#### 2.1.2. UCI HAR

The dataset was created with the help of 30 participants aged 19 to 48 years old, who used smartphones (Samsung Galaxy SII) strapped to their waists [26]. Walking, rising and descending stairs, sitting, standing, and lying down were all actions performed by each participant. The data-collecting process occurred at a constant frequency of 50 Hz, aided by the smartphone’s embedded gyroscope and accelerometer. The initial dataset included nine attributes derived from body acceleration, total acceleration, and angular velocity along three axes. The signals were processed using a median filter. The dataset yielded 561 characteristics and had 10,299 samples. The dataset’s activity distribution is depicted in Figure 2b.

### 2.2. Data Preprocessing

A consistent preprocessing method was applied to the two datasets used in the experiment. Initially, the various types of raw data files were consolidated into a single HDF5 file with a defined data structure. The datasets were then divided into training, testing, and validation sets using Scikit-learn’s train-test split function, maintaining a 70:15:15 ratio. Due to the substantial size of the original data, which complicates direct processing by the classification model, segmentation was necessary during preprocessing. The segmentation length chosen for the experiment was 128. The data were read using a sliding window approach with 50% overlap. Additionally, to account for varying sampling frequencies across the datasets, a downsampling method was employed to standardize the frequencies, making them easier to compare.

To address the class imbalance in the WISDM dataset, we first applied an undersampling technique and normalized the dataset values to a range of 0–1. While undersampling generally improves balance, random oversampling proved ineffective. We explored alternative oversampling techniques, including SMOTE (synthetic minority oversampling technique) and ADASYN (adaptive synthetic sampling). SMOTE, a widely used method designed to enhance random oversampling, improved minority class representation but introduced synthetic noise. ADASYN slightly enhanced minority class detection but also increased false positives. Our findings also indicate that while SMOTE and ADASYN improved minority class representation, they introduced artificial data and increased inference time.

After evaluating these approaches, we adopted a hybrid strategy—combining undersampling for majority classes with slight oversampling for minority classes. The hybrid approach also failed to yield significant improvements. In contrast, undersampling demonstrated the best balance between accuracy and execution speed, making it the preferred approach despite the loss of some majority class data. Figure 2c represents the activity distribution of WISDM data after adopting the undersampling strategy. Additionally, we used standard scaling and transformed categorical features into numerical representations using the leave-one-out encoder.

### 2.3. Machine Learning Classifiers

We employed nine machine learning approaches to identify human activities based on activity data during this experiment. We used random forest (RF), logistic regression (LR), gradient boosting (GBoost), k-nearest neighbor (KNN), extreme gradient boosting (XGB), decision tree (DT), support vector machine (SVM), adaptive boosting (AdaBoost), and multilayer perceptron (MLP) algorithms to recognize six distinct human activities using activity features. LR is frequently used in classification and predictive analytics [27]. It calculates the likelihood of an occurrence, such as voting or not voting, depending on a set of distinct variables. As the outcome is expressed as a probability, the conditional variable is constrained within the range of 0–1. The subsequent classifier utilized is DT, a supervised learning method employed for both classification and regression tasks. DT functions as a tree-structured classifier, with each leaf node denoting the classification outcome, internal nodes representing dataset features, and branches illustrating the decision-making process [28].

Next, we employed a supervised learning technique known as k-nearest neighbor (KNN), which utilizes most of the k-nearest neighbor category to classify the outcomes of new instance queries [29]. This algorithm is one of the most commonly used pattern recognition algorithms. It aims to classify a new item based on its attributes and training data. The k-nearest neighbor method utilizes neighborhood categorization to predict the value of the new query instance. We also experimented with random forest (RF) as a classifier. It is an effective supervised machine learning technique for carrying out tasks related to classification. RF classifiers have numerous parameter values that require tuning. Our primary attention was on two parameters: random-state and n-estimator.

However, we also apply some boosting algorithms. These algorithms merge weak learners into effective learners through an iterative process [30]. GBoost is a boosting classification technique that builds a model by iteratively adding decision trees. Another ensemble model is XGBoost, where high-performance parallel tree boosting is available for classification. It is used to train and boost a single model from a dataset simply. An incremental strategy is used here. It employs a single model succession instead of training all of the models, with the new model being taught to rectify the inaccuracy of the prior model [31]. Among different values of the regularization parameter of the XGBoost model, random_state = 3, learning_rate = 0.05, alpha = 10, gamma = 2, max_depth = 10, and n_estimators = 100 worked best in our case.

We also experimented with another classifier, multilayer perceptron (MLP), alongside each feature extractor. In MLP, an input dimension is transformed to the desired dimension via fully connected dense layers where numerous layers in a neural network are used. The outputs of some neurons become the inputs of other neurons when we join neurons to form a neural network [32]. Last but not least, we employed a support vector machine (SVM), which has a greater accuracy. For the identification of tasks, SVM [33] is used to generate the soundest hyperplane that separates two or more classes.

### 2.4. Deep Learning Classifiers

Four distinct deep learning (DL) classifiers were mainly taken into consideration for feature extraction: CNN, LSTM, CNN-BiLSTM, and Attention-CNN-BiLSTM. The suggested approach’s imposition of the ensemble model leads to utilizing these DL networks. The deep learning networks needed to distinguish between different model outputs to effectively utilize the ensemble model with the most efficient selective deep learning classifiers. Each of these models receives a feature matrix created from the raw sensor data. All of these models have been used on the two distinct datasets (UCI-HAR and WISDM). We will go to great lengths to explain each part in the following sections, describing its roles and contributions to our suggested model.

#### 2.4.1. CNN Recognition Model

We use two one-dimensional convolutional layers with the ReLu activation function to learn discriminant characteristics from sensor data. A brief explanation is: C(32)-C(64)-P, where C(Ls) represents a convolutional layer with Ls feature maps and P represents a max pooling layer. A filter w is used in convolution to create a new feature by applying it to a window of embedded characters in h. For instance, a feature $m_{i}$ is generated by Equation (1)

(1) $$\begin{array}{c} x_{1:n}=x_{1}⊕x_{2}⊕x_{3}⊕...⊕x_{n}\\ m_{i}=f\left(w\cdot x_{i+k-1+b}\right)\\ m=[m_{1},m_{2},...m_{n-k+1}] \end{array}$$

in this equation, *x* represents the embedded domain name vector, *w* denotes the filter, *b* stands for the bias term, and *f* represents the nonlinear ReLU function. To create a feature map, the filter has been applied to every domain character window that could exist. The largest feature of a set of features, m, is obtained by the most excellent pooling operation, and $m_{i}$ indicates a feature acquired by a convolution operation. While the pooling operation can minimize the number of parameters and avoid overfitting, the convolutional operation provides the advantages of local connection and weight sharing, which can simplify the model.

#### 2.4.2. LSTM Recognition Model

When the time steps in a recurrent neural network’s gradient algorithm are excessively small or large, the network’s gradient can either explode or vanish [34]. To mitigate this problem, LSTM incorporates a gating mechanism to regulate information [35]. It introduces an input gate, forget gate, and output gate to filter out information that is no longer pertinent to the current context [36], thereby extending the information storage time and preserving some prior data. The hidden state existing between the current time step input $X^{t}$ and the previous time step $H^{t}$ serves as the input to the LSTM gate. The output obtained from the entire connection layer is as follows:

(2) $$Input\;gate:I^{t}=\sigma \left(X^{t}W^{xi}+H^{t-1}W^{hi}+b^{i}\right)$$

(3) $$Forgetting\;gate:F^{T}=\sigma \left(X^{t}W^{xf}+H^{t-1}W^{hf}+b^{f}\right)$$

(4) $$Gated\;unit:C^{t}=tanh\left(X^{t}W^{xc}+H^{t-1}W^{hc}+b^{c}\right)$$

(5) $$C^{t}=F^{t}⊙C^{t-1}+I^{t}⊙C^{t}$$

(6) $$Output\;gate:O^{t}=\sigma \left(X^{t}W^{xo}+H^{t-1}W^{ho}+b^{o}\right)$$

where the input gate’s weight matrices are $W^{xi}$ and $W^{hi}$, with bias $b^{i}$, while the forgetting gate’s weight matrices are $W^{xf}$ and $W^{hf}$, with bias $b^{f}$. The candidate memory cells are denoted as $C^{t}$, and the gated unit’s weight matrices are $W^{xc}$ and $W^{hc}$, with bias $b^{c}$. The output gate’s weight matrices are $W^{xo}$ and $W^{ho}$, with bias $b^{o}$. The activation function of the tanh function regulates data transfer within the hidden state through element-wise multiplication. The output gate $O^{t}$ manages the information flow from the memory cell to the hidden state, resulting in the final output $H^{t}$, which is

(7) $$H_{t}=O_{t}⊙tanh\left(C_{t}\right)$$

in contrast to traditional LSTM models, the BiLSTM (bi-directional long short-term memory) approach integrates both forward and backward LSTM components. During feature extraction, it captures holistic information by considering data in both forward and reverse orientations [37]. The outputs from these dual extractions are then combined and aggregated in a specialized manner, leveraging insights from two distinct perspectives. This strategy reduces the input data order’s influence on individual LSTMs’ output, thereby yielding more comprehensive results.

The design essentially comprises three layers: one LSTM layer, one dropout layer, and one dense layer. The LSTM layer has a size of 64. After the LSTM layer, a dropout layer is incorporated. The output layer utilizes the Softmax activation function, and the Adam optimizer and the categorical cross-entropy loss function were employed.

#### 2.4.3. CNN-BiLSTM Recognition Model

While the architecture of CNN-BiLSTM lacks the depth of pre-trained models, its performance is at least on par with state-of-the-art models. Several trials with hyperparameter adjustment were used to construct this model. The hyper-parameters and their chosen attributes are in Table 2. The architecture consists of nine layers, including two convolution, two max pooling, two BiLSTM, one dense, one dropout, and one output layer. The input sizes are 128 × 3 for the WISDM dataset and 128 × 9 for the UCI-HAR dataset. The number of filters for the first and second CNN are 64 and 128, respectively, whereas both BiLSTM layers have 64 filters. Each convolution layer used the ReLU activation function, and for optimization, the categorical cross-entropy loss function and softmax activation function were utilized with the Adam optimizer.

#### 2.4.4. Attention with CNN-BiLSTM Recognition Model

The attention, CNN, BiLSTM, and Attention-CNN-BiLSTM models are used to classify human activities. The proposed models were chosen because of their proven robust performance. The attention technique’s primary objective is to imitate people’s capacity for attention. To understand information, people usually focus on a limited number of essential areas rather than giving all of the information equal attention. The BiLSTM is adequate for processing time series data, CNN for spatial data [38], and CNN-BiLSTM for both. BiLSTM designs have been effectively used in time series [39]. Therefore, incorporating an attention mechanism into the prediction method enables the assignment of different weights to the data. This helps mitigate the effects of irrelevant input data on the output and amplifies the influence of crucial input data. The structure of the attention model is depicted in Figure 1d. See Zheng and Chen [40], for instance, for the precise computation steps. Figure 1c depicts the construction of the Attention-CNN-BiLSTM model. The architecture consists of 10 layers, including 1 attention, 2 convolution, 2 max pooling, 2 BiLSTM, 1 dense, 1 dropout, and 1 output layer. The input sizes are 128 × 3 for the WISDM and 128 × 9 for the UCI-HAR. The number of filters for the first and second CNN are 64 and 128, respectively, whereas both BiLSTM layers have 64 filters. Each convolution layer used the ReLU activation function, and for optimization, the categorical cross-entropy acts as a loss function and softmax as an activation function, along with the Adam optimizer.

### 2.5. Selecting the Most Effective Classifiers and Techniques for Extracting Features

**Figure 3:**
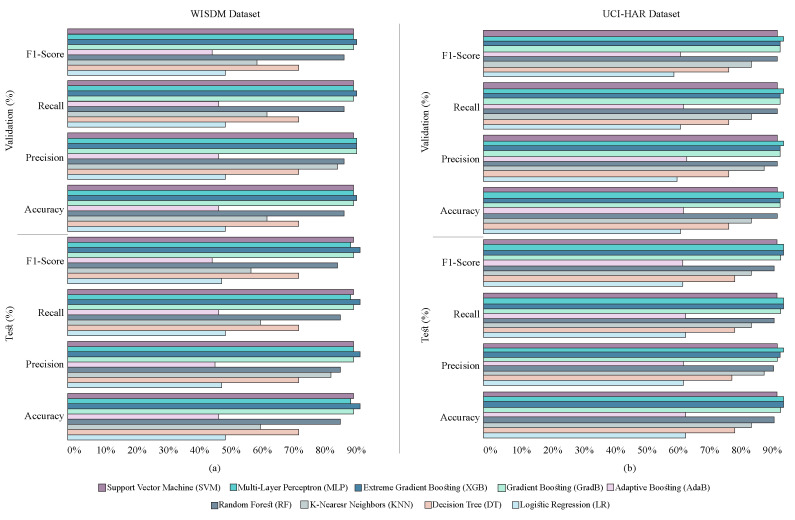
Accuracy-based efficiency of ML algorithms and feature extractors. RF, GradB, XGB, MLP, and SVM achieved the highest average accuracy among all classifiers when utilizing the (**a**) WISDM dataset and (**b**) UCI-HAR dataset.

**Figure 4:**
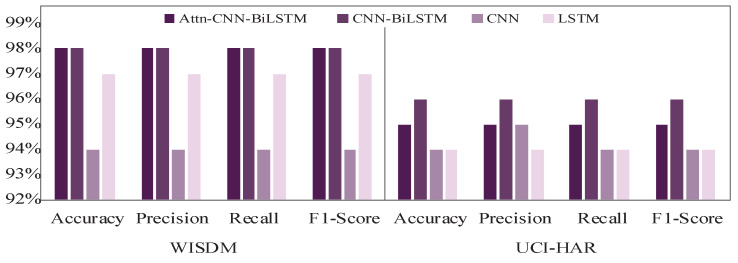
Accuracy-based performance of DL classifiers and feature extractors. Cnn-BiLSTM and Attention-Cnn-BiLSTM achieved the highest average accuracy among all classifiers when utilizing the WISDM and UCI-HAR dataset.

Using the feature extractors for the datasets WISDM and UCI-HAR, nine distinct machine learning (ML) and four distinct deep learning (DL) classifiers have been tested to determine the best classifier. Figure 3a,b indicate the average accuracy, precision, recall, and f1-score for the test and validation sets of all ML classifiers. The four performance matrices considering the two datasets are shown here. RF, GradB, XGBoost, MLP, and SVM were selected for additional classification tests because it is evident from Figure 3a,b that they beat all other ML classifiers. However, out of all the DL classifiers, attention-based CNN and BiLSTM (AttnCnnBiLSTM) and CnnBiLSTM performed the best, as shown in Figure 4. Subsequently, using datasets WISDM and UCI-HAR, all of the feature extractors (CNN, LSTM, CnnBiLSTM, and AttnCnnBiLSTM) are tested.

Thus, the most effective feature extractors are selected based on their efficacy on distinct datasets. For building the ensemble model, the proposed AttnCnnBiLSTM and CnnBiLSTM are identified as the top feature extractors.

### 2.6. Proposed Model: Activity Identification Using Suggested Three-Stage Ensemble Network

In this work, the feature-extracting procedure is done in three phases. The strategies that fared well in the first two stages were selected for the ensemble.

#### 2.6.1. Feature Level Ensemble in the First Stage

Out of the nine ML classifiers that were primarily taken into consideration for this work, the most promising models were selected in the first step. The suggested methods RF, GradB, XGB, MLP, and SVM, respectively, were determined to have the best accuracy for the WISDM and UCI-HAR datasets, as Figure 3 illustrates. Therefore, these five models have been selected for the initial stage ensemble out of the nine models. With an average accuracy improvement, the first_stage ensemble approach performed significantly better compared with most of the individual models.

#### 2.6.2. Feature Level Ensemble in the Second Stage

According to Figure 4, two DL models—AttnCnnBiLSTM and CnnBiLSTM—are qualified for the second-stage ensemble based on the validation accuracy. The approach has also been implemented using GRU (gated recurrent unit), and the results are identical to LSTM. LSTM often has more parameters than GRU owing to the additional gate (forget gate). This can make LSTM more powerful, but it also increases the likelihood of overfitting, particularly on smaller datasets. GRU has fewer settings because it lacks a forget gate. This can make it more computationally efficient and resistant to overfitting, making it an excellent alternative for smaller datasets. However, studies have shown that LSTM can outperform GRU, achieving up to 2% higher accuracy in certain scenarios [41]. Because the dataset we utilized contained a significant number of samples, we tested the performance using trial and error, and the results were practically equal. Considering the used dataset, LSTM was chosen over GRU. The second-stage ensemble framework is then constructed by further concatenating the two models that were chosen.

#### 2.6.3. Third-Stage Feature Level Ensemble: Attention-CNN-BiLSTM and Selective-ML-Based Human Activity Recognition Model

Unlike previous classification models, Attention-CNN-BiLSTM and CNN-BiLSTM can take into account all information embedded in input data while also emphasizing the importance of key input features. Moving from two-dimensional data processing enables these models to produce more precise results, as they are better suited to capture complex data structures. Our approach applies a three-stage ensemble method, the stages of which are as follows. Stage 1 involves a selective ensemble of ML classifiers (RF, GradB, XGBoost, MLP, and SVM), which reduces model complexity and prevents overfitting, providing a strong foundation for robust initial predictions. Stage 2 utilizes the Attention-CN-BiLSTM and CNN-BiLSTM ensemble to refine predictions by capturing complex sequential patterns in the data and highlighting essential features through the attention mechanism, enhancing recognition accuracy. In stage 3, weighted average aggregation occurs, where the final weights are assigned through a step-by-step optimization process, allowing the ensemble to adaptively emphasize predictions with the highest reliability and thus enhance the overall accuracy and robustness of HAR outcomes. In this stage, we use a weighted average to aggregate predictions from the previous two ensembles (stage 1 and stage 2) to produce a balanced output. The weight for each ensemble output is calculated based on the accuracy and F1-score of each model on a validation set, allowing us to emphasize models with higher robustness. This weighted aggregation helps to mitigate the biases or weaknesses inherent in any single approach, thus improving the model’s overall generalizability and robustness. By combining diverse strengths, the three-stage ensemble minimizes recognition errors and ensures high accuracy across various activity types. The framework is represented in Figure 1b.

## 3. Evaluation and Analysis

This section determines and discusses the performance of the created activity recognition algorithm. The efficiency of the proposed method is verified through Python simulation. We compare first-, second-, and third-stage ensembles and consider three benchmark models, XGBoost, CNN, and LSTM, since they are significant to our method in this research.

### 3.1. Evaluation Metrics

The performance of the proposed model is assessed using several different statistical metrics, including accuracy coefficient, precision, recall, and F-measure. The most used performance indicator for assessing a binary classification model is accuracy. It calculates the percentage of all predictions the model made that were accurate. The precision metric quantifies the percentage of actual positive cases among the instances the model predicts to be positive. The ratio of true positives to the sum of true positives and false negatives is referred to as recall. The harmonic mean of recall and precision is known as the F1-score.

(8) $$\begin{array}{c} Accuracy=(TP+TN)/(TP+FP+FN+TN) \end{array}$$

(9) $$\begin{array}{c} Precision=TP/(TP+FP) \end{array}$$

(10) $$\begin{array}{c} Recall=TP/(TP+FN) \end{array}$$

(11) $$\begin{array}{c} F1-Score=2\times (precision\times recall)/(precision+Recall) \end{array}$$

The variables in Equations (8)–(11) represent the quantity of true positive (TP), true negative (TF), false positive (FP), and false negative (FN).

### 3.2. Parameter Settings of Used DL and ML Classifiers

The parameter settings for DL and ML classifiers for the proposed model are shown in Table 2 and Table 3.

#### 3.2.1. Parameter Settings of Deep Learning Models

The primary factors that affect how accurate the Attention-CNN-BiLSTM and CNN-BiLSTM are were essentially determined by criteria such as the number of units, input feature dimensions, hidden layer states, layer count, width of hidden layers, and training iterations. Table 2 contains the particular parameter settings for the Attention-CNN-BiLSTM and CNN-BiLSTM models.

#### 3.2.2. Parameter Settings of Different ML Classifiers

The performance of the RF, GradB, XGBoost, MLP, and SVM models is primarily influenced by several factors including the number of decision trees, the model’s training progress, the sample size used for random sampling with replacement, iterative decision tree techniques, choice of weak learner, objective function used by these machine learning algorithms, regularization parameters, and complexity control. The parameter configurations for these selected machine learning models are outlined in Table 3.

## 4. Result and Discussion

Our three-stage ensemble model, “Attention-CNN-BiLSTM with selective ML” (Model 6), demonstrates competitive results by outperforming recent HAR models in both accuracy and generalizability. Garcia-Gonzalez [14] achieved robust results with ML on smartphone data but lacked ensemble diversity, an aspect our model addresses with selective ML. Meanwhile, Yalcinkaya [18] and Minowa [23] concentrated on collaborative and incomplete data handling, respectively, though their models showed limitations in adaptability. Our weighted ensemble approach, confirmed through an ablation approach, maintained high F1-scores across datasets, outperforming simpler DL and ML methods, and thereby validating its robustness for complex activity recognition. For our analysis, we have included three benchmark models and two ensemble models (Models 1–5), with our proposed “three-stage ensemble” model as Model 6. Table 4 provides an overview of these six models, and the comparative results between Model 6 and the others are detailed in Table 5 and Table 6. Our model emerged as the most efficient in recognition accuracy. Due to the imbalanced nature of the UCI-HAR and WISDM datasets, relying solely on accuracy for a meaningful comparison was limited. Therefore, we incorporated the F1-score alongside accuracy to evaluate the recognition efficacy comprehensively. This dual-metric approach further underscores the adaptability and effectiveness of Model 6, confirming its superior performance across diverse datasets and scenarios.

**Figure 5:**
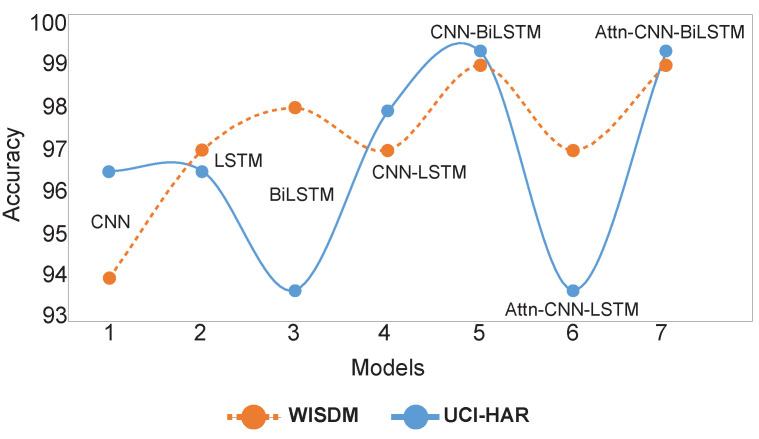
Graphically representing the comparative analysis of the benchmark model with various combinations, including CNN, LSTM, BiLSTM, CNN-LSTM, CNN-BiLSTM, Attn-CNN-LSTM, and Attn-CNN-BiLSTM.

We also conducted an ablation study to validate the contributions of each model component, testing the following configurations: CNN, LSTM, BiLSTM, CNN-LSTM, CNN-BiLSTM, Attention-CNN-LSTM, and Attention-CNN-BiLSTM. These models were evaluated on the WISDM and UCI-HAR datasets to determine their relative effectiveness for human activity recognition (HAR). The ablation study confirms that the CNN-BiLSTM and Attention-CNN-BiLSTM configurations deliver the highest accuracy and F1 scores on both datasets, indicating that combining CNN with bidirectional LSTM and attention mechanisms enhances the model’s ability to capture complex temporal dependencies in HAR data. Figure 5 visually represents the performance of each model, underscoring the superiority of CNN-BiLSTM and Attention-CNN-BiLSTM considering both the F1 score and accuracy measurements.

### 4.1. Impact of Using Three-Stage Ensemble Model

**Figure 6:**
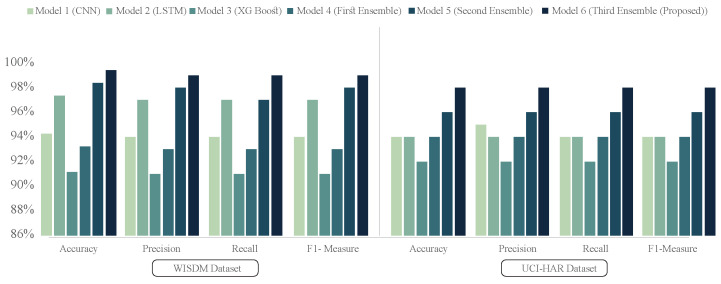
Comparative analysis of different models (Model 1 to Model 6).

**Figure 7:**
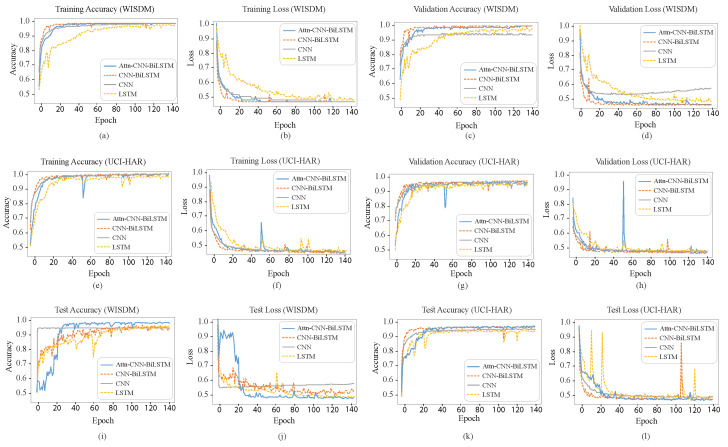
Impact of DL models on WISDM and UCI-HAR dataset: epochs vs. training, validation and test accuracy and loss for CNN, LSTM, CNN-BiLSTM, and Attention-CNN-BiLSTM. Figures (**a**,**c**,**i**) illustrate the training, validation, and testing accuracy of the WISDM dataset, while (**b**,**d**,**j**) depict its training, validation, and testing loss. Similarly, figures (**e**,**g**,**k**) represent the training, validation, and testing accuracy of the UCI-HAR dataset, whereas (**f**,**h**,**l**) show its training, validation, and testing loss.

It is relatively rare for a single CNN network to extract every significant characteristic from the input data. It is impossible to attain excellent generalization capabilities when training a single model on a single dataset. This research employs a three-stage ensemble strategy to alleviate this restriction. The performance boost of the three-stage ensemble model is evident in Figure 6, where the accuracy of the WISDM and UCI-HAR datasets are shown separately. Figure 6 depicts a comparison of the recognition accuracy of CNN, LSTM, XGBoost, first ensemble (RF, GradB, SVM, MLP, XGBoost), second ensemble (CNN-BiLSTM, Attention-CNN-BiLSTM), and third ensemble (first-stage ensemble + second-stage ensemble) combined models. We discovered that our proposed network has the best recognition accuracy. The average accuracy of the first-stage ensemble using the WISDM dataset is 92.93%. Before applying the third-stage ensemble, we see that the improvement in the framework’s average accuracy is incorporated after the second-stage ensemble by 5.56% to 98.49%. Finally, following the third-stage ensemble, the suggested model’s average accuracy is 99.58%. As a result, the overall accuracy improves by 6.65%. The accuracies of the first-, second-, and third-stage ensembles for the UCI-HAR dataset are 94.75%, 96.57%, and 98.75%, respectively. It is demonstrated that the model ensemble’s accuracy improves by nearly 4%. As we can see, average accuracy improves for three-stage ensembles for WISDM and UCI-HAR. The accuracies and loss curves for each DL network on the WISDM and UCI-HAR datasets are illustrated in Figure 7.

Our proposed model, “Attention-CNN-BiLSTM with selective ML”, has significant potential for medical diagnosis and patient monitoring applications. By accurately recognizing human activities and differentiating subtle patterns, it supports critical healthcare needs, particularly for individuals with disabilities or chronic conditions. In the medical diagnosis field, its ability to classify and monitor activities with high precision is encouraging in diagnosing movement-related disorders, such as Parkinson’s disease, arthritis, or musculoskeletal impairments. For example, by analyzing sensor data from wearable devices, the system can detect abnormal gait patterns, tremors, or deviations in daily activity routines, providing early indicators of health issues. Such capabilities offer clinicians a data-driven approach to diagnosis, enabling them to base medical decisions on objective, real-time activity metrics rather than subjective observations alone.

Continuous activity monitoring is critical for managing patients with disabilities or those undergoing rehabilitation. The ensemble framework’s robustness against sensor noise and overlapping activities ensures reliable monitoring, even in complex or dynamic environments. For disabled individuals, the model can detect changes in activity levels or unusual behaviors, alerting caregivers or healthcare providers for timely intervention. This functionality is particularly vital for fall detection or inactivity monitoring, reducing the risk of undetected medical emergencies in remote or home care settings. The incorporation of weighted aggregation further enhances the model’s applicability in healthcare by addressing imbalanced datasets, which are common in medical scenarios where rare but critical activities (e.g., falls) are less frequent than routine movements. This ensures that the system remains sensitive to high-risk activities while maintaining overall performance. Finally, by leveraging advanced ensemble methods, this study may contribute to the growing field of personalized medicine. The model adapts to individual variations in activity patterns, supporting tailored healthcare plans for rehabilitation, fitness tracking, or chronic disease management. Additionally, its scalability and adaptability make it suitable for integration with Internet of medical things (IoMT) platforms, enabling seamless data sharing between patients, caregivers, and medical professionals.

### 4.2. Verifying the Robustness and Generalizability of the Model

A profound classification model must have a high degree of generalization capacity. An inability to generalize renders a model unsuitable for practical implementation. Various experiments have been conducted to verify the resilience and generalization capabilities. This includes k-fold cross-validation. The first-stage ensemble model is validated using k-fold cross-validation in the following experiment, proving its durability. Since it makes use of every observation in the training and validation sets, it is a more suitable method for validating a model [42]. The dataset is divided into k equal sections for training and testing purposes. This guarantees that no observation is made without first being tested by the model. The size of the dataset affects k’s value. For every dataset, we used a 10-fold cross-validation process, and Table 7 depicts the results of each fold.

### 4.3. Computational Complexity and Inference Time

To assess the trade-offs between accuracy and computational efficiency, we conducted a complexity analysis comparing our three-stage ensemble model with simpler architectures, such as a single CNN and a single BiLSTM. The results demonstrate that while the single CNN model exhibited the lowest inference time, approximately 0.821 ms in UCI-HAR and 0.407 ms in WISDM per sample, it struggled to differentiate complex human activities, limiting its overall classification performance. In contrast, the BiLSTM model required a higher number of floating-point operations (FLOPs), resulting in an inference time of approximately 5.104 ms in UCI-HAR and 5.272 ms in WISDM per sample. However, it demonstrated improved sequential modeling capabilities, making it more suitable for recognizing temporal dependencies in human activities. The key findings are summarized in Table 8.

Our proposed three-stage ensemble model, although computationally more expensive with an inference time of approximately 5.8 and 3.6 ms per sample in UCI-HAR and WISDM, respectively, achieved significantly better classification accuracy, outperforming the simpler models by 2–4% in terms of F1-score. The increased computational cost is justified by the model’s enhanced robustness and generalizability, which are crucial for real-time healthcare monitoring and rehabilitation applications. By leveraging the strengths of multiple architectures, our approach ensures a more reliable and adaptive human activity recognition system, making it well-suited for medical informatics applications where precision is critical.

### 4.4. Comparative Analysis of WISDM Dataset

Table 9 shows an accuracy comparison of the suggested approach alongside various existing networks for the WISDM dataset. The suggested network outperforms earlier research. The reason for this is the spatial extraction of features carried out by the CNN and BiLSTM layers and the selective ML models, which enhanced the performance of the Attention-CNN-BiLSTM with selective ML models.

**Figure 8:**
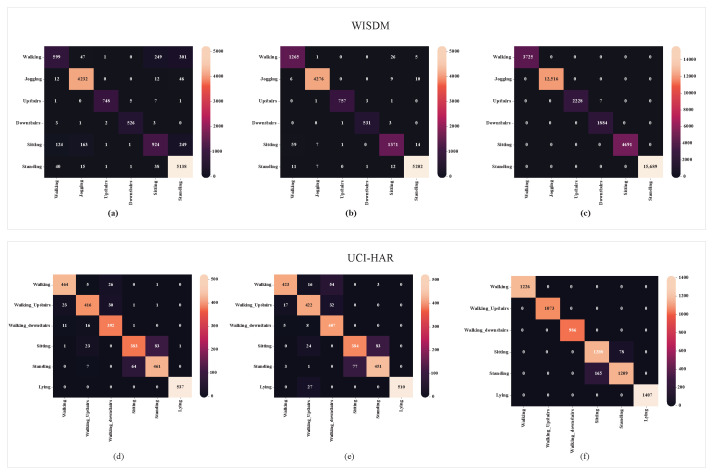
Confusion matrix of the (**a**) first-stage ensemble model, (**b**) second-stage ensemble model, and (**c**) third-stage ensemble model (proposed) of the WISDM dataset; and (**d**) first-stage ensemble model, (**e**) second-stage ensemble model, and (**f**) third-stage ensemble model (proposed) of the UCI-HAR dataset.

The confusion matrix (CM) obtained on the WISDM dataset is depicted in Figure 8a–c. It reveals that the three-stage ensemble model achieves greater convergence during training.

### 4.5. Comparative Analysis of UCI HAR Dataset

Table 10 compares the efficacy of the suggested approaches with the performance of the existent models [16,22,25,36,44,45,46,48] in terms of F1-score and/or accuracy. Figure 8d–f show the confusion matrix (CM) from the UCI-HAR dataset. Experiments were conducted to compare the suggested approach against established approaches, including CNN-LSTM, CNN-BiLSTM, and CNN-GRU. However, they achieved the highest mean recognition rate of 98%. The findings show that the recommended model outperforms existing HAR comparison methodologies.

### 4.6. Validation with PAMAP2 Dataset

To better evaluate the effectiveness of the proposed methods, we also perform experiments using the PAMAP2 dataset [49], which includes complex activities. The dataset consists of 18 daily physical activities (including 6 optional ones) recorded for 9 participants (1 female and 8 males), with activity names and distribution. The data comprise readings from accelerometers, gyroscopes, magnetometers, temperature sensors, heart rate monitors, and other devices placed on the subjects’ hands, chest, and ankles, totaling 54 dimensions and sampled at 100 Hz. Of the data dimensions, 12 irrelevant to the study were discarded in the experiment, leaving 42 dimensions only for further analysis. For the final classification, 6 optional activities were excluded, focusing on identifying and classifying 12 daily activities. The selected activities and their associated numerical labels are as follows: 1: lying, 2: sitting, 3: standing, 4: walking, 5: running, 6: cycling, 7: nordic walking, 12: ascending stairs, 13: descending stairs, 16: vacuum cleaning, 17: ironing, and 24: rope jumping. These activity labels reflect a range of dynamic and static movements, enabling comprehensive evaluation of activity recognition algorithms. The numbering scheme originates from the original dataset structure, ensuring consistency with prior studies using PAMAP2.

**Figure 9:**
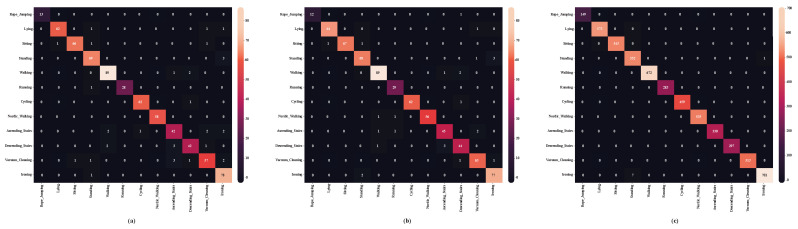
Confusion matrix of the (**a**) first-stage ensemble model, (**b**) second-stage ensemble model, and (**c**) third-stage ensemble model (proposed) of the PAMAP2 dataset.

Figure 9 presents the normalized confusion matrices for the first, second, and third ensembles, achieving average accuracies of 95%, 96%, and 99%, respectively. In contrast, traditional methods like CNN, LSTM, and CNN-LSTM did not exceed 95% accuracy. This highlights the robustness of the proposed approach, demonstrating superior performance on the PAMAP2 dataset.

### 4.7. Comparison with Existing Work

Recent advancements in HAR have leveraged various deep learning models and hybrid approaches to improve activity recognition, particularly in the healthcare sector. Conventional HAR methods, such as CNN and LSTM, have shown efficacy in capturing spatial and temporal patterns within activity data [50]. However, these models often lack mechanisms for prioritizing key data features, which can be crucial for refining accuracy in complex activities. Models like CNN-LSTM hybrids and attention-enhanced networks have recently emerged as powerful solutions, achieving notable improvements by introducing a focus on critical input patterns, particularly in continuous monitoring scenarios [51,52]. Our model advances these approaches by combining an attention mechanism, bi-directional LSTM (BiLSTM), and selective machine learning classifiers. Introducing attention layers enhances the model’s ability to assign appropriate weights to critical data points, improving interpretability and recognition accuracy. By integrating machine learning models, our model optimizes BiLSTM further, creating a robust, ensemble-based solution that surpasses the predictive performance of CNN, LSTM, CNN-LSTM, and other benchmark models [13]. The comparative performance on the UCI-HAR and WISDM datasets illustrates our model’s superiority, achieving 98.75% and 99.58% accuracy, respectively, outperforming other advanced HAR frameworks.

## 5. Conclusions

This study introduces a novel three-stage ensemble model, “Attention-CNN-BiLSTM with selective ML”, specifically designed to advance human activity recognition (HAR) with a focus on healthcare applications for disabled individuals. Unlike traditional HAR models, which often rely on single-stage architectures or lack adaptability, our approach uniquely integrates attention mechanisms, CNN, BiLSTM, and selective ML algorithms into a unified framework. This innovative ensemble strategy, coupled with a weighted aggregation method optimized iteratively, significantly enhances recognition accuracy, robustness, and generalizability across diverse activities and datasets. Through a comprehensive ablation study and benchmarking on WISDM and UCI-HAR datasets, we demonstrate the superiority of our model compared to state-of-the-art methods, highlighting its ability to address critical challenges such as imbalanced datasets, sensor noise, and overlapping activities. Furthermore, the tailored design of the model for healthcare applications showcases its real-world relevance, enabling precise activity monitoring and rehabilitation support for disabled individuals. By addressing gaps in traditional HAR systems and pushing the boundaries of ensemble-based approaches, this research contributes not only to the advancement of HAR methodologies but also to their impactful applications in medical informatics. The proposed model sets a foundation for scalable and adaptive solutions, emphasizing the importance of innovative frameworks in achieving reliable and versatile activity recognition as well as diagnosis and prognosis of different health conditions.

## Figures and Tables

**Table 1:** Details of two public datasets used in this study.

Datasets	Used Sensors	Activities	Participants	Sampling Freq.	Samples
WISDM	Gyroscope	6	36	20 Hz	1,086,474
UCI-HAR	Accelerometer, gyroscope	6	30	50 Hz	10,299

**Table 2:** Setting of parameters of Attention-CNN-BiLSTM and CNN-BiLSTM.

Meaning of the Parameter	Parameter	Value of the Parameter
Total no. of units	unit	128
Time step	time-step	32
The no. of unit layers	num-layers	2
Width of the hidden layer	batch-size	512
No. of iteration	epochs	150
Activation function (input)	activation	ReLU
Activation function (output)	activation	Softmax
Optimizer	optimizer	Adam
Loss function	loss	categorical-crossentropy

**Table 3:** Setting of parameters of five different ML classifiers.

Meaning of the Parameter	Parameter	Value of the Parameter
Total no. of decision trees	n-estimators	100
The utmost depth of the decision tree	max-depth	10
Iterative decision tree	learning-rate	0.05
Regularization parameter	alpha	10
Management of model complexity	gamma	2

**Table 4:** Five different models with the proposed one.

Model	Description
Model 1	CNN
Model 2	LSTM
Model 3	XGBoost
Model 4	First ensemble
Model 5	Second ensemble
Model 6	Third ensemble (proposed)

**Table 5:** Benchmark models, other models, and proposed model performance metrics on the WISDM dataset.

Models	Accuracy (%)	Precision (%)	Recall (%)	F1-Measure (%)
Model 1 (CNN)	93.84	93.87	93.84	93.85
Model 2 (LSTM)	96.88	96.92	96.88	96.88
Model 3 (XGBoost)	90.77	90.62	90.77	90.57
Model 4 (First Ensemble)	92.93	92.88	92.93	92.88
Model 5 (Second Ensemble)	98.45	98.46	98.49	98.47
Model 6 (Third Ensemble (Proposed))	99.58	99.57	99.58	99.57

**Table 6:** Benchmark models, other models, and proposed model performance metrics on the UCI-HAR dataset.

Models	Accuracy (%)	Precision (%)	Recall (%)	F1-Measure (%)
Model 1 (CNN)	95.21	95.22	95.21	95.20
Model 2 (LSTM)	94.95	95.06	94.95	94.93
Model 3 (XGBoost)	92.42	92.48	92.43	92.44
Model 4 (First Ensemble)	94.75	94.79	94.76	94.76
Model 5 (Second Ensemble)	96.57	96.58	96.57	96.57
Model 6 (Third Ensemble (Proposed))	98.75	98.71	98.74	98.72

**Table 7:** Assessment of the initial ensemble model following a ten-fold cross-validation process.

Dataset	Fold1 (%)	Fold2 (%)	Fold3 (%)	Fold4 (%)	Fold5 (%)	Fold6 (%)	Fold7 (%)	Fold8 (%)	Fold9 (%)	Fold10 (%)
WISDM	82.11	83.94	87.15	82.56	81.65	85.35	80.27	82.48	78.80	81.56
UCI-HAR	87.74	89.67	85.16	88.38	89.67	91.55	87.01	87.66	82.46	87.66

**Table 8:** Analysis of inference time per sample in the WISDM and UCI-HAR datasets.

Model	WISDM		UCI-HAR	
	Accuracy (%)	Inference Time (ms)	Accuracy (%)	Inference Time (ms)
CNN	93.84%	0.407	95.21%	0.821
BiLSTM	98.85%	5.272	92.10%	5.104
CNN-BiLSTM	99.36%	2.591	96.31%	3.129
XGBoost	90.77%	0.030	92.42%	0.076
**Proposed**	99.58%	3.611	98.75%	5.826

**Table 9:** Evaluation of the performance of the proposed model against existing approaches on the WISDM dataset.

Years	Models	F1-Measure (%)	Accuracy (%)	Precision (%)	Recall (%)
2020	LSTM-CNN [43]	95.19%	95.85%	94.75%	95.65%
2021	Attention-induced multi-head CNN [36]	98.37%	98.18%	97.89%	98.87%
2021	Multi-input CNN-GRU [44]	96.79%	97.21%	96.35%	97.24%
2022	Multi-branch CNN-BiLSTM [45]	95.54%	96.05%	95.08%	96.01%
2022	CNN-based Bi-LSTM parallel model with attention [46]	95.85%	95.86%	95.85%	95.85%
2023	MI-1D-CNN, feature fusion method [25]	83.68%	84.65%	84.52%	82.87%
2023	Attention-mechanism-based deep learning [22]	93.89%	93.95%	93.87%	93.90%
2024	WISNet [47]	96.31%	96.41%	96.66%	95.97%
-	**Proposed**	98.58%	99.58%	98.59%	98.58%

**Table 10:** Evaluation of the performance of the proposed model against existing approaches on the UCI-HAR dataset.

Years	Models	F1-Measure (%)	Accuracy (%)	Precision (%)	Recall (%)
2021	Multi-input CNN-GRU [44]	96.02%	96.20%	95.89%	96.17%
2021	Attention-induced multi-head CNN [36]	95.57%	95.38%	95.89%	95.27%
2020	CNN-LSTM [48]	91.88%	92.13%	91.79%	91.98%
2022	Multi-branch CNN-BiLSTM [45]	96.13%	96.37%	95.99%	96.28%
2022	CNN-based Bi-LSTM parallel model with attention mechanism [46]	96.14%	96.71%	96.19%	96.11%
2022	Ensem-HAR [16]	95.52%	95.05%	95.09%	95.97%
2023	MI-1D-CNN, feature fusion method [25]	96.88%	97.81%	96.92%	96.85%
2023	Attention-mechanism-based deep learning [22]	93.52%	93.48%	93.09%	93.97%
2024	WISNet [47]	95.38%	95.66%	95.29%	95.47%
-	**Proposed**	97%	98%	96%	97%

## Data Availability

No new data were created or analyzed in this study. The following publicly archived datasets were analyzed during the study: https://archive.ics.uci.edu/datasets (accessed on 23 February 2020).
